# Cardiac Tamponade Caused by Campylobacter ureolyticus Purulent Effusion

**DOI:** 10.7759/cureus.56051

**Published:** 2024-03-12

**Authors:** Michael Obregon, Ahmed Khan

**Affiliations:** 1 Internal Medicine, Southern Illinois University School of Medicine, Springfield, USA; 2 Infectious Diseases, Southern Illinois University School of Medicine, Springfield, USA

**Keywords:** obstructive shock, septic shock, cardiac tamponade, pericardial effusion, campylobacter ureolyticus

## Abstract

A male in his 60s presented to the emergency department with a seven-day history of progressively worsening malaise, dyspnea, nausea, and vomiting. The patient quickly developed septic and obstructive shock, with the ensuing investigation significant for a purulent pericardial effusion causing cardiac tamponade. Subsequent cultures grew *Campylobacter ureolyticus,* which is commonly associated with the gastrointestinal tract and is one of many microorganisms that cause diarrhea*.* Yet, studies have identified this pathogenic organism in oral infections, infectious meningitis, and soft tissue infections, but not pericardial effusions. This organism is an emerging pathogen and warrants renewed research efforts.

## Introduction

*Campylobacter ureolyticus* (*C. ureolyticus*) is a gram-negative, non-spore-forming, anaerobic, aflagellate bacillus that was first described in the literature in 1948. This bacterium was initially called* Bacteroides corrodens *(*B. corrodens*), named in part, due to its appearance on agar, which was described as “pitting” or “corroding” [1]. Further work isolated the bacteria from buccal abscesses that further solidified *B. corrodens* as a newly established bacterium [2]. Yet, as microbiological techniques improved, *B. corrodens* was further separated into several species. For example, *B. corrodens* and *Eikinella corrodens* were separated based on chemotherapeutic agents, the latter being a strict anaerobe and the former being a facultative anaerobe [3]. In 1978, certain strains of *B. corrodens* were further defined, and *Bacteroides ureolyticus* was established based on growth on specific agars, visual spectroscopy of cytochromes, guanine-plus-cytosine content, and lack of pili on electron micrographs [4]. Finally, in 2010, *B. ureolyticus* was reclassified into the *Campylobacter* genus, based on 6S rRNA and cpn60 gene sequences, among other metabolic and cellular fatty acid composition similarities. Thus, *B. ureolyticus* became known as *C. ureolyticus *[5].

## Case presentation

A male in his 60s with a past medical history significant for Charcot-Marie-Tooth disease with peripheral neuropathy, hypertension, hyperlipidemia, and coronary artery disease status post percutaneous intervention with intracoronary stent placement in the 1990s, presented to a local emergency department (ER) with complaints of 7-10 days of worsening malaise, dyspnea, nausea, and vomiting. The patient had a longstanding history of chronic non-healing foot ulcers, which had progressively worsened over the span of a month prior to his ER visit. The patient was hypoxic with a pulse oximeter of 80% on room air and hypotensive with systolic blood pressure in the 60s. The patient received three 1-liter boluses of normal saline but ultimately was started on intravenous (IV) norepinephrine. Due to increasing norepinephrine requirements, the patient was eventually started on epinephrine, vasopressin, and dopamine. The patient underwent rapid sequence intubation due to hypoxia. Initial lab work was significant for abnormal troponin, lactic acid, b-type natriuretic peptide, white blood cell count, serum creatine (baseline 0.9 mg/dL), serum bicarbonate, and arterial blood gas pH (see Table 1).

**Table 1:** Significant lab work on presentation to the emergency room and tertiary medical center

Test name	Results (emergency room)	Results (tertiary medical center)	Lab reference range
Arterial blood gas pH	7.33	6.9	7.35-7.45
Alanine aminotransferase	46 U/L	5,520 U/L	10-60 U/L
Aspartate aminotransferase	47 U/L	13,370 U/L	10-42 U/L
B-type natriuretic peptide	1070 pg/ml	-------------------------------	0-100 pg/ml
Pro-B-type natriuretic peptide	-------------------------------	20,315 pg/ml	<125 pg/ml
Serum bicarbonate	15 mmol/L	8.5 mmol/L	21-32 mmol/L
Serum creatine	3.9 mg/dL	4.21 mg/dL	0.5-1.4 mg/dL
Lactic acid	9.9 mg/dL	16.9 mmol/L	0.4-2.0 mmol/L
Serum potassium	4.3 mmol/L	6.5 mmol/L	3.5-5.5 mmol/L
Troponin	12.33 ng/ml	------------------------------	0.1-0.5 ng/mL
White blood cell count	65.0 x10'3/uL	------------------------------	4.8-10.8 x10'3/uL

**Figure 1:**
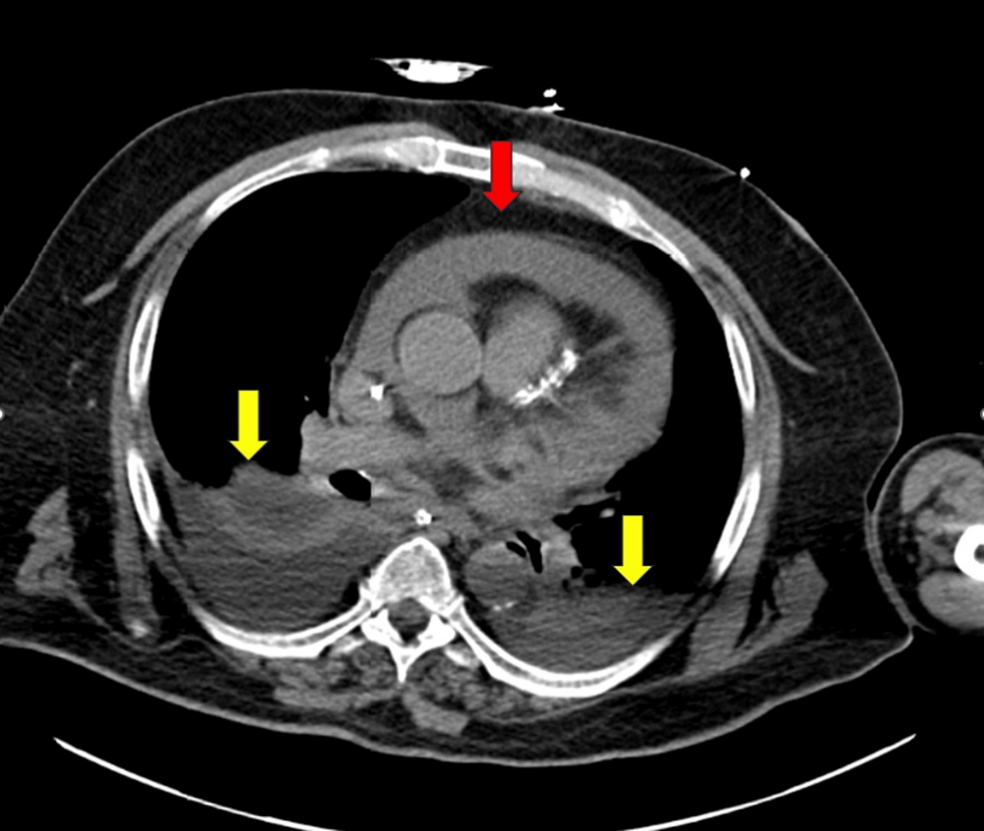
CT chest demonstrating pericardial effusion (red arrow), bilateral pleural effusions (yellow arrows)

An electrocardiogram was significant for atrial fibrillation with a heart rate of 107. CT of the head without contrast was negative for acute pathology. CT chest, abdomen, and pelvis with IV contrast demonstrated a moderate to large pericardial effusion, small bilateral pleural effusions, and bilateral bibasilar atelectasis of the lungs. No acute pathology was noted in the abdomen or pelvis on CT imaging (Figure 1).

On examination, the patient’s right foot appeared dusky, but with palpable dorsalis pedis and bilateral posterior tibial artery pulses. Blood cultures were obtained, which ultimately were negative for growth after five days of incubation. The patient was administered IV vancomycin, piperacillin-tazobactam, and clindamycin. A continuous heparin infusion was initiated for concern for a non-ST elevation myocardial infarction.

**Figure 2:**
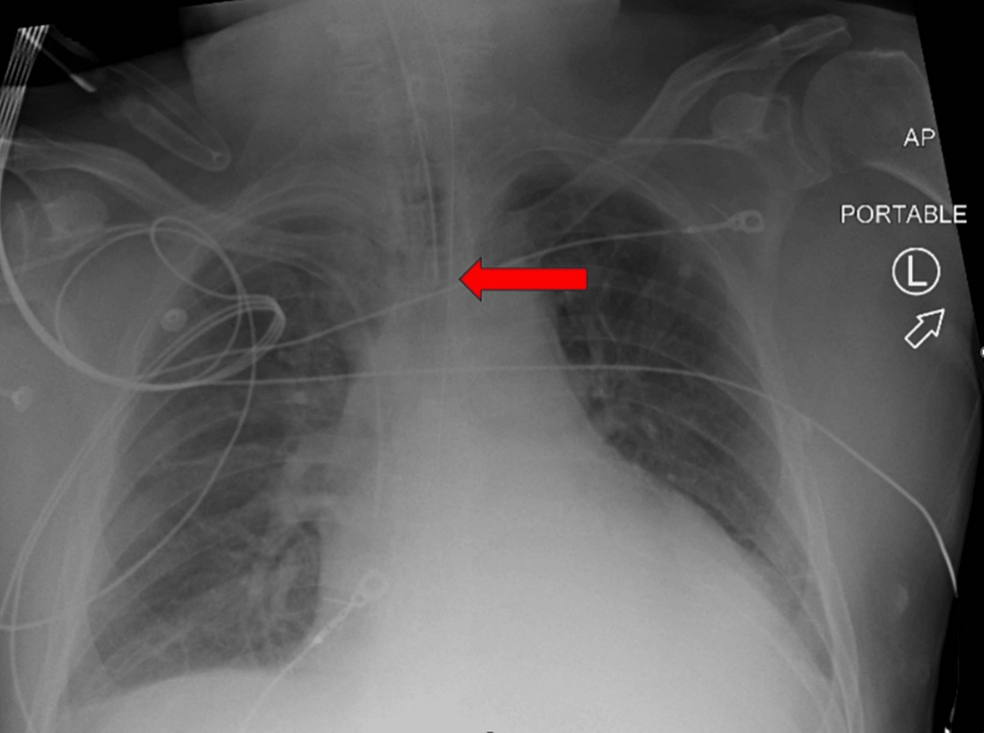
Chest X-ray upon arrival at the tertiary center (red arrow highlighting the end of endotracheal tube)

**Figure 3:**
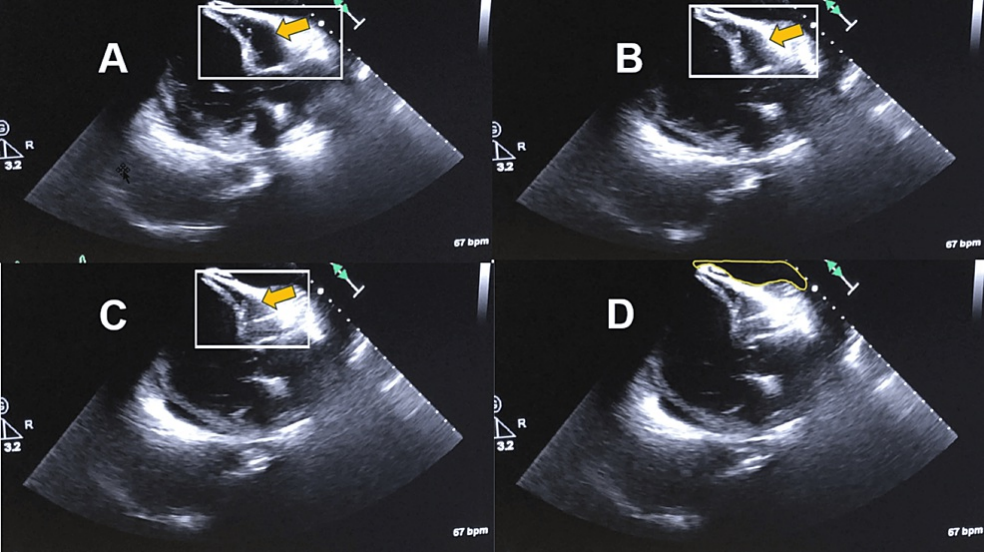
Echocardiogram (off-plane long axis view) with near complete right ventricle collapse (A, B, C) and surrounding pericardial effusion (D)

**Figure 4:**
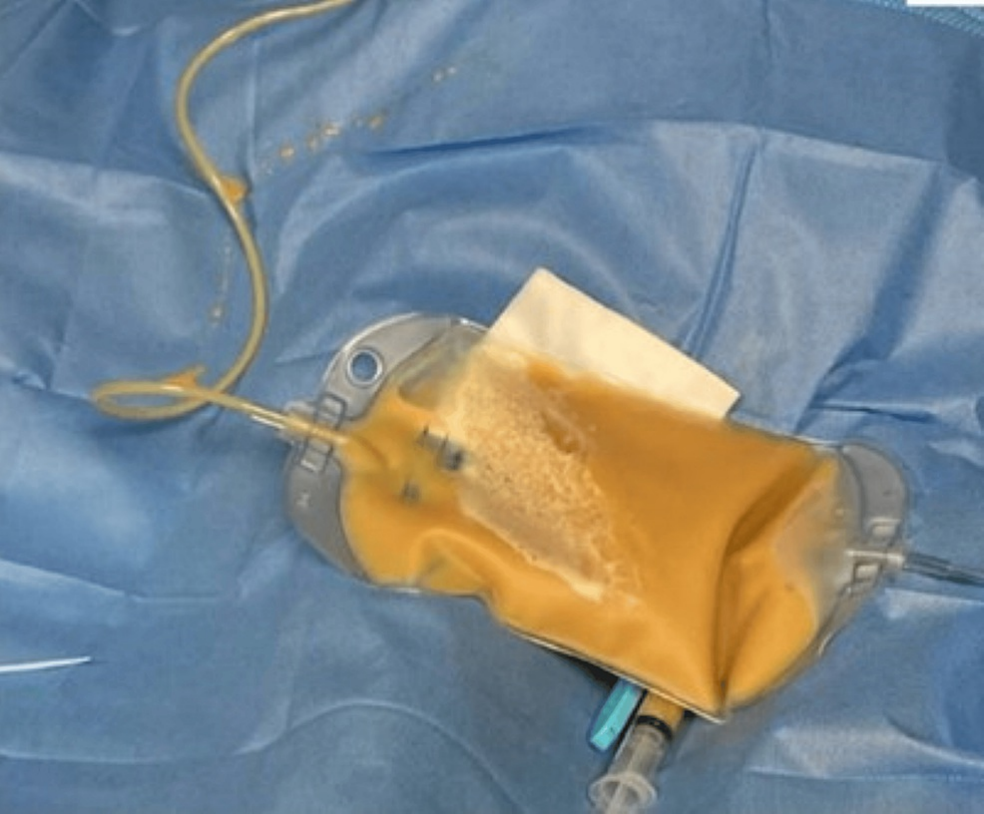
Purulent pericardial effusion

The patient was airlifted to a tertiary medical center for a higher level of care. Labs at the tertiary medical center were repeated (see Table 1). Chest X-ray was repeated to ensure proper endotracheal tube position post-transportation (Figure 2). Cardiology completed an emergent echocardiogram, which demonstrated a left ventricle ejection fraction of less than 15% with tamponade physiology (Figure 3). Bedside pericardiocentesis removed 300cc of purulent fluid (Figure 4). Additionally, the continuous IV heparin infusion was stopped, as the troponins were likely elevated secondary to a type 2 myocardial infarction. 

Nephrology initiated continuous renal replacement therapy (CRRT) for acute kidney failure in the setting of anuria, hyperkalemia, and severe metabolic acidosis. Examination of the left lower extremity had a single 1 cm x 1 cm circular ulcer on the plantar aspect of the foot, which had a heavy callous over the fourth metatarsal head. There was no erythema, drainage, or fluctuance, but the ulcer was positive for probe-to-bone testing. The right lower extremity contained a gangrenous and necrotic fifth toe with an extension of dusky-appearing tissue to the fifth metatarsal on the dorsum and plantar surfaces of the foot. Severe cyanosis with dusky appearing necrosis over the entire dorsum of the forefoot and midfoot, and encroachment onto the hindfoot was observed. The skin over the gangrenous right fifth toe was boggy and fluctuant. Vascular surgery took the patient to the operating room (OR) for right foot debridement with possible amputation due to concern for gangrene and to achieve source control of the infection. In the OR, much of the right forefoot was noted to be necrotic, and a small abscess was encountered lateral to the fifth metatarsal. He underwent debridement of the right foot.

Infectious disease service continued IV clindamycin and vancomycin. Piperacillin-tazobactam was discontinued due to the patient’s acute kidney failure, and he was transitioned to IV cefepime. Several additional cultures were obtained. Blood cultures grew Prevotella species (sensitive to ampicillin-sulbactam, cefoxitin, clindamycin, meropenem, metronidazole, moxifloxacin, penicillin, and piperacillin-tazobactam) and *Staphylococcus capitis* (sensitive to gentamicin, rifampin, tetracycline, trimethoprim-sulfamethoxazole, and vancomycin). Tissue culture obtained from the patient’s foot debridement grew coagulase-negative *Staphylococcus *species, *Citrobacter* species, and *Providencia rettgeri*. Anaerobic culture of the pericardial fluid grew *C. ureolyticus*.

The morning after the patient’s arrival, the patient suffered a cardiac arrest. Advanced cardiovascular life support was initiated and the family was updated, who decided to stop resuscitation.

## Discussion

*C. ureolyticus* has been implicated in a range of infections including non-gonococcal urethritis, soft tissue infections, oral infections, and most notably as an emerging gastrointestinal disease [6-9]. The main reservoir of *C. ureolyticus* appears to be in poultry, typically associated with the farming industry, but has also been isolated from unpasteurized or raw milk [10]. Disease transfer is typically through the fecal-oral route, and this pathogen is considered a foodborne pathogen. For example, one study by Bullman et al. [11] found that *C. ureolyticus* was found in 41% of stool samples from patients suffering from diarrheal illness in Southern Ireland, which was second to *Campylobacter jejuni*, found in 50.7% of stool samples. Similar studies echo the theme that *C. ureolyticus* may be more prevalent than previously thought throughout the world. Increasing incidence, though may be related to improved culture and molecular techniques [11-15].

*C. ureolyticus* has several virulence factors that aid in the colonization and development of invasive diseases in humans and animals. This pathogen, in higher quantities, will produce an exopolymeric matrix that aids in adherence to cellular tissues [16]. In a separate study, Bullman et al. [17] reported on proteins that enabled *C. ureolyticus* to adhere, invade, and secrete toxins that facilitate translocation across the intestinal cellular barrier, such as tight junctions. Just as work has been done to elucidate virulence factors, so has work been completed on identifying antibiotics that are effective at treating *C. ureolyticus* infections. Antibiotics like penicillin, amoxicillin-clavulanate, piperacillin-tazobactam, cefoxitin, cefotetan, ceftriaxone, clindamycin, imipenem, meropenem, metronidazole have all been found to be effective [18].

Aside from being implicated as the causative pathogen in a higher percentage of infectious causes of diarrhea, *C. ureolyticus* has become a more clinically relevant pathogen. For example, Mukhopadhya et al. [19] reported a higher prevalence of *C. ureolyticus* in colonic biopsies in adults with ulcerative colitis, which may suggest that *C. ureolyticus* plays a role in chronic inflammation of the large intestine. In the acute setting, Prevel et al. [20] identified a difference in the biodiversity of patients that had a higher incidence of mortality among critically ill patients in the ICU. *C. ureolyticus* was one such organism associated with the non-survivor group.

Our case illustrates an unusual cause of pericardial purulent effusion, which represents the first case of such to our knowledge. While *C. ureolyticus* is increasingly recognized as a gastrointestinal pathogen, its involvement in non-gastrointestinal pathology, particularly pericardial effusions, remains exceptionally uncommon. Most studies and clinical reports focus on its association with infections such as non-gonococcal urethritis, soft tissue infections, and oral infections. The distinctiveness of this case prompts a reevaluation of the spectrum of infections associated with *C. ureolyticus*, emphasizing the need for heightened clinical awareness and further research into the diverse manifestations of this emerging pathogen. As the medical community continues to encounter rare presentations of known pathogens, such as in this case, the importance of comprehensive microbial investigations and understanding the broader clinical impact of these organisms becomes increasingly paramount in providing optimal patient care.

## Conclusions

Campylobacter ureolyticus is an emerging pathogen, commonly associated with gastrointestinal infections, which is being noted to cause more invasive disease. Obstructive shock may not be apparent on the initial assessment of a patient. Bedside point-of-care ultrasound provides a useful tool to quickly identify abnormal pathology and lead to appropriate confirmatory testing. Patients with a complex medical history, with rapidly progressing severe symptoms, emphasize the importance of considering a broad differential diagnosis. 

## References

[1] Henriksen SD (1948). Studies in gram-negative anaerobes. I. A hemophilic gram-negative rod. APMIS.

[2] EI M (1958). Studies on an anaerobic, rodshaped, gram-negative microorganism: Bacteroides corrodens n. sp. Acta Pathol Microbiol Scand.

[3] Robinson JV, James AL (1974). In vitro susceptibility of Bacteroides corrodens and Eikenella corrodens to ten chemotherapeutic agents. Antimicrob Agents Chemother.

[4] Jackson FL, Goodman YE (1978). A new species to accommodate strains previously identified as “Bacteroides corrodens, anaerobic”. Int J Syst Bacteriol.

[5] Vandamme P, Debruyne L, De Brandt E, Falsen E (2010). Reclassification of Bacteroides ureolyticus as Campylobacter ureolyticus comb. nov., and emended description of the genus Campylobacter. Int J Syst Evol Microbiol.

[6] Hawkins DA, Fontaine EA, Thomas BJ, Boustouller YL, Taylor-Robinson D (1988). The enigma of non-gonococcal urethritis: role for Bacteroides ureolyticus. Genitourin Med.

[7] Duerden B, Bennet KW, Faulkner J (1982). Isolation of Bacteroides ureolyticus (B corrodens) from clinical infections. J Clin Pathol.

[8] Lee S, Lee J, Ha J (2016). Clinical relevance of infections with zoonotic and human oral species of Campylobacter. J Microbiol.

[9] Bullman S, Corcoran D, O'Leary J, Lucey B, Byrne D, Sleator RD (2011). Campylobacter ureolyticus: an emerging gastrointestinal pathogen?. FEMS Immunol Med Microbiol.

[10] O'Donovan D, Corcoran GD, Lucey B, Sleator RD (2014). Campylobacter ureolyticus: a portrait of the pathogen. Virulence.

[11] Bullman S, O'Leary J, Corcoran D, Sleator RD, Lucey B (2012). Molecular-based detection of non-culturable and emerging campylobacteria in patients presenting with gastroenteritis. Epidemiol Infect.

[12] Basic A, Enerbäck H, Waldenström S, Östgärd E, Suksuart N, Dahlen G (2018). Presence of Helicobacter pylori and Campylobacter ureolyticus in the oral cavity of a Northern Thailand population that experiences stomach pain. J Oral Microbiol.

[13] Hatanaka N, Shimizu A, Somroop S (2017). High prevalence of Campylobacter ureolyticus in stool specimens of children with diarrhea in Japan. Jpn J Infect Dis.

[14] Serichantalergs O, Ruekit S, Pandey P, Anuras S, Mason C, Bodhidatta L, Swierczewski B (2017). Incidence of Campylobacter concisus and C. ureolyticus in traveler's diarrhea cases and asymptomatic controls in Nepal and Thailand. Gut Pathog.

[15] Kaakoush NO, Castaño-Rodríguez N, Mitchell HM, Man SM (2015). Global epidemiology of Campylobacter infection. Clin Microbiol Rev.

[16] Burgos-Portugal JA, Kaakoush NO, Raftery MJ, Mitchell HM (2012). Pathogenic potential of Campylobacter ureolyticus. Infect Immun.

[17] Bullman S, Lucid A, Corcoran D, Sleator RD, Lucey B (2013). Genomic investigation into strain heterogeneity and pathogenic potential of the emerging gastrointestinal pathogen Campylobacter ureolyticus. PLoS One.

[18] Roberts SA, Shore KP, Paviour SD, Holland D, Morris AJ (2006). Antimicrobial susceptibility of anaerobic bacteria in New Zealand: 1999-2003. J Antimicrob Chemother.

[19] Mukhopadhya I, Thomson JM, Hansen R, Berry SH, El-Omar EM, Hold GL (2011). Detection of Campylobacter concisus and other Campylobacter species in colonic biopsies from adults with ulcerative colitis. PLoS One.

[20] Prevel R, Enaud R, Orieux A (2022). Gut bacteriobiota and mycobiota are both associated with Day-28 mortality among critically ill patients. Crit Care.

